# Heterogeneous ensemble approach with discriminative features and modified-SMOTEbagging for pre-miRNA classification

**DOI:** 10.1093/nar/gks878

**Published:** 2012-09-24

**Authors:** Supatcha Lertampaiporn, Chinae Thammarongtham, Chakarida Nukoolkit, Boonserm Kaewkamnerdpong, Marasri Ruengjitchatchawalya

**Affiliations:** ^1^Biological Engineering Program, King Mongkut’s University of Technology Thonburi, Bang Mod, Thung Khru, Bangkok, 10140, ^2^Biochemical Engineering and Pilot Plant Research and Development Unit, National Center for Genetic Engineering and Biotechnology at King Mongkut’s University of Technology Thonburi, Bang Khun Thian, Bangkok, 10150, ^3^School of Information Technology, King Mongkut’s University of Technology Thonburi, Bang Mod, Thung Khru, Bangkok, 10140, ^4^School of Bioresources and Technology and ^5^Bioinformatics and Systems Biology Program, King Mongkut’s University of Technology Thonburi, Bang Khun Thian, Bangkok, 10150, Thailand

## Abstract

An ensemble classifier approach for microRNA precursor (pre-miRNA) classification was proposed based upon combining a set of heterogeneous algorithms including support vector machine (SVM), k-nearest neighbors (kNN) and random forest (RF), then aggregating their prediction through a voting system. Additionally, the proposed algorithm, the classification performance was also improved using discriminative features, self-containment and its derivatives, which have shown unique structural robustness characteristics of pre-miRNAs. These are applicable across different species. By applying preprocessing methods—both a correlation-based feature selection (CFS) with genetic algorithm (GA) search method and a modified-Synthetic Minority Oversampling Technique (SMOTE) bagging rebalancing method—improvement in the performance of this ensemble was observed. The overall prediction accuracies obtained via 10 runs of 5-fold cross validation (CV) was 96.54%, with sensitivity of 94.8% and specificity of 98.3%—this is better in trade-off sensitivity and specificity values than those of other state-of-the-art methods. The ensemble model was applied to animal, plant and virus pre-miRNA and achieved high accuracy, >93%. Exploiting the discriminative set of selected features also suggests that pre-miRNAs possess high intrinsic structural robustness as compared with other stem loops. Our heterogeneous ensemble method gave a relatively more reliable prediction than those using single classifiers. Our program is available at http://ncrna-pred.com/premiRNA.html.

## INTRODUCTION

MicroRNAs (miRNAs) are small endogenous non-coding RNAs (≈19–25 nt). They play crucial roles in post-transcriptional regulation of gene expression of plants and animals (1). The miRNAs are expressed at different levels during cell proliferation, metabolism, development, apoptosis and tumor metastasis (1–2). In animals, miRNA biogenesis begins with the transcription of several-hundred-nucleotides-long primary transcripts called primary miRNAs (pri-miRNAs). An enzyme called Drosha recognizes hairpin substructures in the pri-miRNAs and cleaves them to produce ∼70-nt long miRNA stem-loop precursors (pre-miRNAs) (3). The pre-miRNAs are then subsequently processed to yield mature miRNA by Dicer enzyme, which targets pre-miRNAs on the basis of their hairpin secondary structures, which are considered as a crucial characteristic for enzyme substrate recognition in miRNA biogenesis pathways (4). A number of miRNAs remain undiscovered. Identification of miRNA genes is one of the most imminent problems towards the understanding of post-translational gene regulation in both normal development and human pathology (5).

There are two main approaches in miRNA identification: experimental and computational approaches. The discovery and characterization of novel miRNA genes have proved to be challenging both experimentally and computationally (6). Experimental approaches have successfully identified highly expressed miRNAs from various tissues. However, cloning methods are biased towards miRNAs that are abundantly expressed (3,5,7). Computational methods have been developed to complement experimental approaches in facilitating biologists for identifying putative miRNA genes. These methods offer the most cost-effective and time-effective screening approaches to identifying miRNAs. There are two types of computational techniques: comparative and non-comparative methods. The former is based on identifying conservation of sequences from closely related species to find homologous pre-miRNAs. However, a key drawback of this approach is their lack of ability to detect novel pre-miRNAs that are not homologous to previously identified miRNAs. For the latter, classification models are trained by machine learning (ML) in identifying non-conserved miRNAs, both known and novel, based on miRNA characteristics. Numerous *de novo* non-comparative methods for identifying pre-miRNA hairpins based on single ML algorithm have been proposed (8–16). For such methods, stem-loop structures are involved in prediction. However, the stem-loop structures of non-miRNA sequences, similar to those of pre-miRNAs, can be found all over the genome. This could lead to a high false positive rate (FPR). Moreover, there is a risk of over-fitting of an algorithm to the training data. Therefore, the computational *de novo* method should be improved to obtain a more efficient and reliable pre-miRNA classification method. To handle the false positive and the over-fitting, we introduced an ensemble technique in ML to the problem of pre-miRNA classification. The ensemble, the committee of various algorithms, has been known to provide more reliable and less false positive results than a single classifier through the agreement among heterogeneous classifiers. Each single algorithm has its own strengths (and weaknesses) depending on the induction hypothesis embedded in its learning process; no single algorithm can perform significantly better than others in all problems and performance measurements (17–20). The voting of distinct algorithms can reduce the bias occurring in a single learning algorithm and this can therefore be relatively more generalized in prediction on new unseen data. (18,21–23). Performances of ensemble ML-based methods have been examined extensively (24–30) and they have been proven to be effective in various applications, such as optical character recognition, face recognition, protein classification and gene expression analysis (18,31–32).

In general, most ML-based methods rely on known pre-miRNA characteristics as features for training prediction models. Among these specific features, hairpin secondary structure and minimum free energy (MFE) of stem-loop hairpins are considered as key features (4). However, plant pre-miRNAs have been reported to have different characteristics from those of animals in MFE distribution, size and stem-loop structure (3,14,33). Moreover, MFE of hairpin structure was not a unique characteristic for miRNA because some small non-coding RNA (ncRNA) also has high negative MFE value similar to those of pre-miRNAs (34). It has been reported that the stem loops of pre-miRNAs exhibit a significantly high level of genetic robustness in comparison with other stem-loop sequences (35–37). The high intrinsic robustness of miRNA stem loops which goes beyond the intrinsic robustness of other stem-loop structures is likely a consequence of selection for functionally relevant substructure toward increased robustness (38). In this study, we considered various robustness features of miRNA such as Z-score, *P*-value and self-containment (SC) score. The SC score is an *in silico* used to measure the structural robustness property of the RNAs in the face of perturbations. It has been shown that both plant and animal pre-miRNA hairpins have particularly high SC scores, with right-skewed distribution, compared with other hairpins. Since the pre-miRNAs need to maintain stable structural folding through cleavage steps during their biogenesis pathway, the pre-miRNA stem loops exhibit high SC whereas pseudo-hairpin sequences and other structured RNAs do not (39). Therefore, we were interested in exploring these kinds of robustness characteristics of pre-miRNAs.

In addition, there are two challenging issues for further enhancement of ensemble performance. Firstly, irrelevant and redundant features can significantly reduce the performance of classifiers. Therefore, identification of discriminatory features is required. Secondly, for training data, the class of interest (minority class) is rare and has less data than the majority class, which is commonly found in Bioinformatics data, including pre-miRNA data. In the case of imbalanced data, algorithms aim to maximize overall accuracy and bias toward the majority class. Thus, rebalancing the imbalanced training data is a necessary step for improving performance on both sensitivity and specificity.

This study presents a novel heterogeneous ensemble combining various efficient classifiers to the problem of pre-miRNA classification. The method, a cooperative combination of different learning algorithms exposed to different training subsets, can create a high level of diversity and reduce bias that tends to occur when single individual classifier is used. Consequently, the ensemble provides a more reliable prediction. Additionally, novel robustness features were introduced: the SC-base pair composite features served as promising discriminators in distinguishing real pre-miRNA hairpins from other hairpin sequences with improved sensitivity and specificity from an original SC feature. Moreover, a feature selection (FS) method was applied to select only relevant and discriminative features. The problem of imbalanced data was solved by the modified-Synthetic Minority Oversampling Technique (SMOTE) bagging method. This enhanced ensemble-based method would effectively differentiate pre-miRNA from non-miRNA sequences with higher accuracy and better balanced sensitivity and specificity score, across various organisms, making our model a useful tool for finding novel animal, plant and virus pre-miRNAs.

## MATERIALS AND METHODS

### Data set

#### Training data

We randomly selected 600 non-redundant sequences of 1424 *Homo sapiens* pre-miRNAs, 200 of 491 of *Oryza sativa*, and 200 of 232 of *Arabidopsis thaliana* from the miRBase version 17 (40) as our positive data sets, where *H. sapiens, O. sativa* and *A. thaliana* represent animal, monocot plant and dicot plant positive data, respectively.

The negative training data set was composed of both pseudo-hairpin sequences and other ncRNAs. 8494 non-redundant pseudo-hairpins were extracted from the protein-coding region (CDS) according to the human RefSeq genes. The pseudo-hairpins were selected based on the following criteria: (i) length distribution of pseudo sequences similar to those of pre-miRNAs, (ii) have a minimum 18 bp on stem structure and (iii) a maximum −18 kcal/mol of free energy. A set of 4000 pseudo-hairpin sequences, randomly selected from 8494 hairpins, were represented as one type of negative training data set. A set of 754 other non-coding RNA (ncRNAs) originally from the Rfam database (41) is another type of negative training data, composed of 327 tRNAs (transfer RNAs), 5 sRNAs (small RNAs), 53 snRNAs (small nuclear RNAs), 334 snoRNAs (small nucleolar RNAs), 32 YRNAs (non-coding RNA components of Ro ribonucleoproteins) and 3 other miscellaneous RNAs. These non-redundant ncRNA sequences have length between 70 and 150 nt and can form hairpin structures. In this work, four independent testing sets were used to evaluate the performance of the algorithm. Description of the four testing data sets is presented in Supplementary Method S1.

### Features

We gathered 103 features previously introduced in other works (8–9,11–12), including 19 sequence-based features, 24 secondary-structure-based features, 28 base-pair features and 32 triplet-sequence-structure-based features.

This study, not only included all features used in previously proposed methods, but also incorporated structural-robustness-related properties into the feature set. We defined new features—namely ‘the SC-derived feature’ which were SC/(1 − dP), SC × dP, SC × dP/(1 − dP), SC/tot_bp, SC/Len, SC × MFE/Mean_dG, SC × zG, SC/NonBP_A, SC/NonBP_C, SC/NonBP_G and SC/NonBP_U—and incorporated them into the list.

The list of 125 features used in our study is summarized in Table 1. Detailed descriptions of these features are provided in Supplementary Method S2.

**Table 1.** List of 125 features used in this work

Feature groups	No. of features	Feature symbol
Sequence-based features	19	Len, %G+C,% A+U, %AA, %AC, %AG, %AU, %CA, %CC, %CG, %CU, %GA, %GC, %GG, %GU, %UA, %UC, %UG, %UU
Secondary structure features	30	MFE, efe, MFEI1, MFEI2, MFEI3, MFEI4, dG, dQ, dD, dF, Prob, zG, zQ, zD, zF, nefe, Freq, diff, dH, dH/L, dS, dS/L, Tm, Tm/L, **MFEI5, MFE/Mean_dG, dH/loop, dS/loop, Tm/Loop, dQ/Loop**
Base pair features	32	dP, zP, div, tot_bp, stem, loop, A-U/L, G-U/L, G-C/L, %A–U/Stem, %G–C/Stem, %G–U/Stem*,* Probpair1–10, Avg_BP_stem, NonBP_A, NonBP_C, NonBP_G, NonBP_U, Non_BPP, **%A–U/BP, %C–G/BP, %G–U/BP, Avg_BP_Loop**
Triplet sequence structure	32	A(((, A((., A(.., A(.(,A.((,A.(.,A..(, A …, C(((, C((., C(.., C(.(, C.((, C.(., C..(, C…, G(((, G((., G(.., G(.(, G.((, G.(., G..(, U…, U(((, U((., U(.., U(.(, U.((, U.(., U..(, U…,
Structural robustness features (SC-derived features)	12	**SC, SC/tot_bp, SC/Len, SC × MFE/Mean_dG, SC × dP, SC × zG, SC/(1 − dP), SC×dP/(1 − dP), SC/NonBP_A, SC/NonBP_C, SC/NonBP_G , SC/NonBP_U**
Total	125	

Our additional features are shown in bold.

### FS methods

We considered three filter FS methods: ReliefF, Information Gain (InfoGain) and correlation-based feature selection (CFS) (42–45). Details of the FS determination are described in Supplementary Method S3.

### Algorithm selection

To select base classifiers for constructing an ensemble, various algorithms were compared. Eight algorithms—naïve bayes (NB), neural networks (MLP), support vector machine (SVM), k-nearest neighbors (kNN), decision tree (J48), repeated incremental pruning to produce error reduction (RIPPER), RBF network (RBFNets) and random forest (RF)—were considered in our algorithm selection experiment. Each displays a different inductive bias and learning hypotheses (instance-based, rules, trees and statistics) and, therefore, provides a potentially more independent and diverse set of predictions to build upon. The details of algorithms are described in Supplementary Method S4.

### Ensemble method

**Figure 1.**
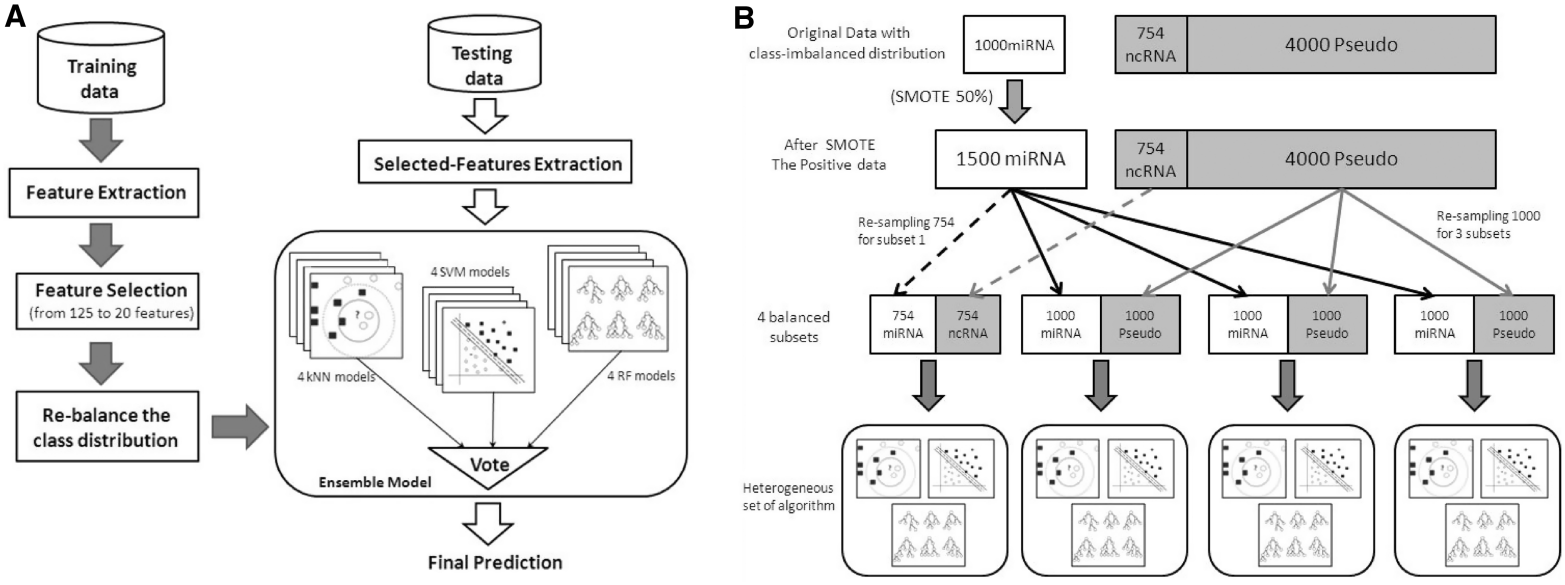
(**A**) Overview of proposed ensemble method: the training process is shown by dark thick arrows. The testing process is shown in white arrows. (**B**) Rebalancing class distribution procedure: the imbalanced training data were processed to obtain four subsets of training data with balanced distribution between positive and negative classes.

Our heterogeneous ensemble method was implemented using Perl and Java scripts. Our program was run on a Fedora Linux-based machine. We used Weka (46), LIBSVM (47,48) and R programming (49) to build and compare base classifiers. The computational procedure of our method was illustrated in Figure 1A. The training process started from collecting positive and negative data. Each sequence in the training data was extracted as an input vector of 125 features by a feature extraction process. Then, the FS method selected informative and relevant features and removed irrelevant and redundant features from the 125 feature set. The sub-sampling methods were applied to rebalance the distribution in the training data as illustrated in Figure1B. To handle the class imbalance in the data set, the resampling techniques, both over-sampling and under-sampling, were integrated to improve the minority class prediction performance, and were performed as follows. First, we applied the SMOTE (50) with a resampling rate of 50% to increase the frequency of the minority class by synthesizing 500 new samples of the minority class (using the parameter k = 5). Under-sampling was applied to create equal class balance in training subsets by under-sampling the majority class with the same number of examples of minority class. These resampling methods were called the ‘modified-SMOTEbagging’ method. The method finally gave four class-balanced training subsets: one subset of ‘miRNA versus ncRNA’ and three subsets of ‘miRNA versus pseudo-hairpin’. After the rebalancing, the chosen algorithms were then trained on each balanced training subset. As a result, 12 base classifiers (4 SVM, 4 RF, 4 kNN) were combined to form the ensemble. Finally, the predictions of 12 individual classifiers, which were 3 algorithms trained on 4 well-balanced distribution training data subsets, were voted to obtain the final prediction.

### Performance evaluation methods

To precisely assess the predictive power of a prediction method and model comparison, we used several performance measurements already applied extensively in the field of Bioinformatics. All the performance measures are defined as: 
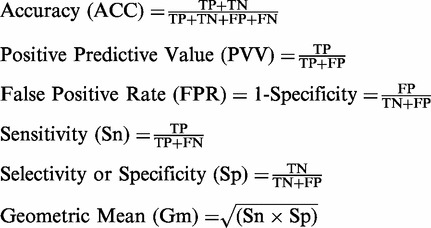



The receiver operating characteristic (ROC) curve is a graphic visualization of the trade-off between the true positive and false positive rates for every possible cut off; we used an area under the ROC curve (AUC) to compare the performance of classifiers.

## RESULTS AND DISCUSSION

### Predictive performance improvement using SC-derivative features

Since the choice of features has an impact on predictive performance of classifier, the discriminative powers for each feature group are compared. The average 5-fold CV (51) performance of different feature groups is shown in the Table 2. The accuracy, commonly used measurement, is not an appropriate metric to evaluate the performance of a classifier in class-imbalanced data since the negative class (majority) in training data is much larger than the positive (minority) class. The geometric mean (Gm) is suitable for evaluating the performance in this situation where class-imbalanced data still occurs because it considers performance on both majority and minority classes (52). Among the five feature groups in this study, the SC derivative group showed the most discriminative power with the highest sensitivity at 84.5%, the highest specificity at 98.4% and the highest Gm at 91.18%. A classifier employing a SC derivative feature group outperformed those employing other feature groups. Moreover, it outperformed the classifier that utilized all 125 features (Gm of 90.86%). This indicates that the SC derivative feature group is a strong discriminant between pre-miRNA and non-miRNA sequences. This result was consistent with previous reports (38–39) in which pre-miRNAs showed high robustness in their structure since pre-miRNAs need to maintain functional structure in the face of perturbation in their biogenesis. The real pre-miRNAs exhibit remarkably high SC, which goes beyond the intrinsic robustness of the stem-loop hairpin structure.

**Table 2.** Predictive performance of each feature groups by the 5-fold CV

Feature groups	No. of features	Sn	Sp	Gm
Sequence features	19	45.0	96.3	65.83
Structure features	30	82.8	97.6	89.90
Base pair feature	32	81.5	97.8	89.28
Triplet sequence structure	32	77.7	97.1	86.86
SC related feature	12	84.5	98.4	91.18
All five feature groups	125	84.0	98.3	90.86
Feature SC	1	76.6	96.9	86.15
Feature SC × dP	1	80.5	98.1	88.86
Feature SC/(1 − dP)	1	81.3	97.9	89.76
Feature SCxdP/(1 − dP)	1	82.2	98.2	89.84
Feature SC × MFE/Mean_dG	1	81.9	97.5	87.76
Feature SC × zG	1	78.9	98.8	89.36
Feature SC/tot_bp	1	0	100	0
Feature SC/Len	1	0	100	0
Feature SC/NonBP_A	1	68.5	98.4	82.09
Feature SC/NonBP_C	1	0	100	0
Feature SC/NonBP_G	1	0	100	0
Feature SC/NonBP_U	1	48.8	98.4	69.29

Sn = Sensitivity, Sp = Specificity and Gm = Geometric mean.

**Figure 2.**
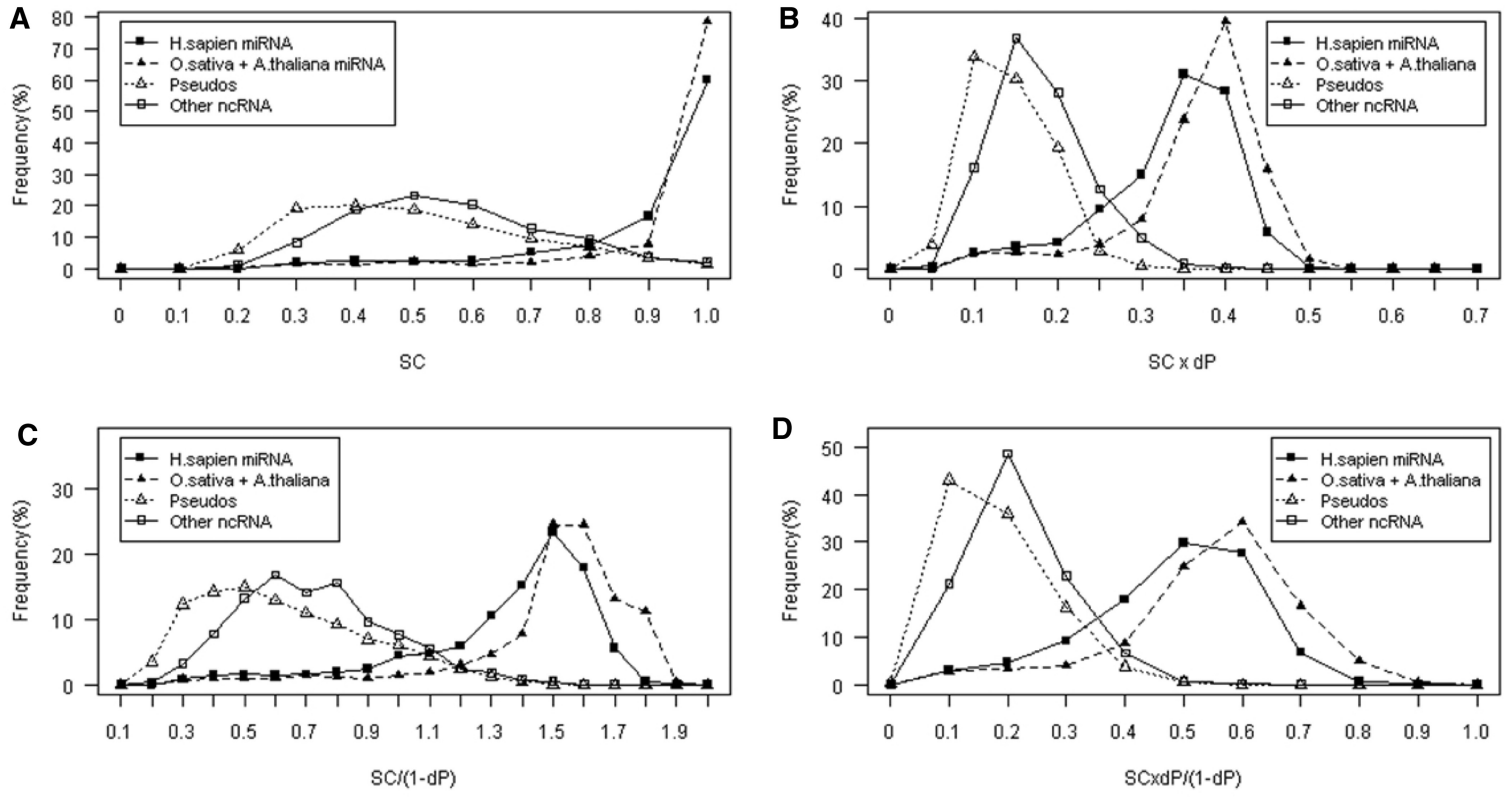
The SC-base pair composite features of Human_miRNA, Plant_miRNA, Other ncRNAs and Pseudo hairpins in our training dataset. (**A**) Original SC feature. (**B**) Feature SC × dP. (**C**) Feature SC/(1 − dP). (**D**) Feature SC × dP/(1 − dP).

Both plants and human pre-miRNAs are similar in SC profile distribution but differ from those of ncRNAs and pseudo-hairpin sequences (Figure 2A). This implies that the SC score can distinguish real pre-miRNAs not only from pseudo hairpin, but also from other small ncRNAs. The result indicated that miRNAs have unique robustness in their structures, which evolved from their functional selection, and this evolved robustness is found in all pre-miRNAs studied in this work. We further investigated this by calculating average SC values in the training data. The average SC values of both human (0.86) and plant (0.91) species are significantly higher than those of other functional RNAs (0.51) and pseudo-hairpin sequences (0.44). This result is consistent with previous studies reporting that pre-miRNA exhibit high intrinsic structural invariance with a strong SC score between 0.85 and 0.98, whereas other stem-loop forming small ncRNAs yield SC ranges (∼0.4–0.6) much lower than the pre-miRNAs (39). Additionally, they observed correlation relationships between SC and various structural features. Thus, these results led to the idea of incorporating various structural features into the SC as our novel features with an aim to maximize specificity and sensitivity.

To further evaluate the performance of individual SC derivative features, the classification performances gained using individual features of our 11 SC-derived features were reported (Table 2). SC-base pair composite features, such as SC × dP, SC/(1 − dP) and SC × dP/(1 − dP), showed the most discriminative features. We found that the use of so-called SC-base pair composite features—the incorporation of information about base pairing or non-base pairing with the SC score—can increase predictive performance by 4–5% in terms of sensitivity value, 1% in terms of specificity and 2–3% in terms of Gm value. By using these three features individually, the classifier distinguishes real pre-miRNA from other hairpins with higher sensitivity and specificity than the original SC score.

In Figure 2B, C and D, the distribution of SC-base pair composite features of real pre-miRNA and negative hairpins were well separated. The human pre-miRNA (*H. sapien*) and plant pre-miRNAs (*O. sativa* and *A. thaliana*) distributions are similar but they differed from those of pseudo-hairpins and other ncRNAs. This result indicated that our SC-base pair combining features were capable of distinguishing real pre-miRNA from other false hairpins across human and plant species. Among the SC-base pair composite features, the feature SC × dP/(1 − dP) yields the highest discriminative power with Gm of 89.84%. The results suggested that using some certain features can give as good performance as using all of the 125 features (Table 2). This may be due to the fact that there are redundant and irrelevant features overall. Therefore, it is reasonably suitable to incorporate the FS method to select only informative, relevant, and non-redundant feature subsets, plausibly increasing the predictive performance of the classifier and decreasing the computation time in the feature extraction process. We investigated three statistical filtering methods based on different criteria, namely ReliefF, InfoGain and CFS + GA. The filter methods for FS rely on general characteristics of data without involving any learning algorithms while the wrapper method needs predetermined classifiers in selecting features. It should be noted that since our method is based on an ensemble system, the wrapper methods that are dependent upon predetermined classifier were not suitable in this study.

To choose the most appropriate FS method, we compared the effectiveness of the 3-fold CV performance of the three filtering methods (Table 3). ‘All features’ and ‘microPred feature’ were also shown as a baseline for comparison. The microPred feature is a set of 21 features from microPred (11), not including our additional features (i.e. SC-derived feature group). In FSs 1 and 2, features were ranked according to ReliefF and infoGain, respectively. The top 75 ranked individual features of the InfoGain criterion produced a Gm of 91.35%. For ReliefF, the top 50 ranked individual features yielded a Gm of 91.40%. The CFS + GA method selected the subset of 20 features with a Gm of 91.49%. The classifiers with selected feature sets (FS1, FS2 and FS3) performed better than classifiers with full feature sets. The possible reasons are some features may be irrelevant and some of them may be redundant due to their high correlation with others in a large number of features. When using the FS method to select relevant and informative features that contribute to discrimination between true and false pre-miRNAs, the performance and robustness of classifiers can be improved (53). Among classifiers with the different FS method, the classifier with the CFS + GA feature set yielded the highest Gm and performed better than those from other methods. Thus, we chose the CFS + GA as a FS method because it gave better overall accuracy and selected a more compact set of features than the other two methods. A selection of relatively fewer features has the advantage of being less time consuming in computing features and increasing the classifier performance by using only informative features. The 20 selected features by the CFS + GA are the following features: Prob, MFEI1, zG, zP, zQ, dH/Loop, Tm/Loop, AU/L, Avg_BP_loop, MFEI5, SC, SC × dP, SC × MFE/Mean_dG, SC × dP/(1 − dP), SC/nonBP_A, Non_BPP, A(((, G(**..**, C **…**and ProbPair4, which were used later in further experiments.

**Table 3.** The average performance by different feature selection algorithms on our training data

Feature subsets	No. of features	Sn (%)	Sp (%)	Gm (%)
All features (No FS)	125	84.0	98.3	90.86
microPred features (J–M) (10)	21	83.0	97.9	90.14
FS1: ReliefF	50	84.9	98.4	91.40
FS2: InfoGain	75	84.9	98.3	91.35
FS3: CFS + GA	20	84.9	98.6	91.49

### Model selection for an efficient ensemble

To select algorithms for construction of an efficient ensemble, various classification algorithms—NB, SVM, kNN, MLP, J48, RIPPER, RBFNets and RF—which have been commonly applied in Bioinformatics, were investigated and compared. Performance of eight different algorithms on the task of pre-miRNA hairpin classification is summarized in Table 4 as the average 10 × 5-fold CV. Among the eight algorithms, SVM, kNN and RF models showed their superior performance in different evaluation metrics. The SVM algorithm gave the highest AUC score on CV. This is likely due to the fact that the algorithm used support vectors that provide a hyperplane with a maximal separation between positive and negative samples, giving the best optimization performance among the eight classifiers. The kNN algorithm yielded the highest specificity and precision measurements of 99.2 and 96.7%, respectively, implying that it performed better in correctly identifying the negative class (false miRNA hairpin sequences) and produced the lowest FPR. The kNN algorithm classified the sample based on the ‘k’ nearest neighbor samples. It produced a satisfactory result for negative data, possibly because negative data have features that are more locally clustered by a closer distance. On the other hand, RF performed most accurately in identifying the positive class (real miRNA hairpin sequences) by yielding the highest sensitivity of 86.7%, similar to previous findings in MiPred (10). This is possibly due to RF, which combined multiple decision trees with multiple discriminative rules that can cover the heterogeneity of characteristics in pre-miRNAs.

**Table 4.** Comparison of the performance of different methods on training data using 20 selected features

Algorithms	Performance measurement					
	ACC	Sn	Sp	PPV	FPR	AUC
K-nearest neighbors (kNN)	95.511	83.3	**99.2**	**96.7**	0.8	0.966
Support vector machine (SVM)	**95.528**	85.1	98.6	94.8	1.4	**0.972**
Artificial neural network (MLP)	95.283	86.5	97.9	92.4	2.1	0.964
Decision tree (J48)	94.581	84.4	97.6	91.3	2.4	0.920
RBF networks (RBFNets)	94.352	86.4	96.7	88.7	3.3	0.968
Rule based (RIPPER)	94.809	84.0	98.0	92.6	2.0	0.923
Naïve bayes (NB)	93.585	85.5	96.0	86.4	4.0	0.955
Random forest (RF)	95.283	**86.7**	97.8	92.1	2.2	0.965

Sn = Sensitivity, Sp = Specificity, PPV = Positive predictive value, ACC = Accuracy, FPR = False positive rate and AUC = Area under ROC curve. The highest values are in bold.

Consistent with the No Free Lunch (NFL) theorem (19), this result strongly suggested that there is no single best algorithm that is superior to all performance metrics. Based on the evaluation, SVM, RF and kNN algorithms were chosen as ensemble members because of their best performances in different metrics: AUC, sensitivity and specificity performance. These three algorithms are different in the way they learn from data. Selecting diverse algorithms will not only combine the strengths of multiple algorithms, but will also make individual classifiers in ensembles disagree with each other. This disagreement among classifiers is utilized by voting to give a reliable final prediction.

### Class-balance and FS enhancing the ensemble performance

In the training data set, pre-miRNA is considered to be a minority class, with the ratio of class distribution being ∼1:5 (miRNA:non-miRNA). It has been shown that the imbalance of pre-miRNAs training data can affect the accuracy of classifiers (11). We performed 10 run of 5-fold CV and investigated the performance of our three different ensemble models in Table 5. Vote1 is the ensemble of three models (SVM, kNN, RF) using all features trained on class imbalance data (original data without performing the resampling techniques). The main difference between Vote1 and Vote2 is the number of features for building models; Vote2 uses 20 features selected from the FS method. Performance can be improved using only relevant and informative features. The ensemble classifiers with the set of selected feature (Vote2) produced better results than the ensemble classifiers with full feature sets (Vote1). By applying FS, we significantly improved the performance of our ensemble from 95.48 to 95.81% in terms of accuracy, and from 0.973 to 0.976 in terms of AUC.

**Table 5.** The 10 × 5 fold CV generalization performance of balanced and imbalanced ensembles with selected features

Algorithms	Performance measurement					
	ACC	Sn	Sp	FPR	Gm	AUC
Vote 1 (Imbalanced, all features)	95.48	84.1	98.7	1.3	91.2	0.973
Vote 2 (Imbalanced, 20 selected features)	95.81	85.1	99.1	0.9	91.4	0.976
Vote 3 (Balanced, 20 selected features)	96.54	94.8	98.3	1.7	96.5	0.996

ACC = Accuracy, Sn = Sensitivity, Sp = Specificity, FPR = False positive rate, Gm = Geometric mean and AUC = Area under the ROC curve.

Unlike Vote1 and Vote2, Vote3 is an ensemble model with 12 classifiers (4 SVM, 4 kNN and 4 RF) trained on class-balanced data, i.e. the SVM, kNN and RF trained on 4 balanced training data subsets (3 × 4 =12). Most ML methods assume the balance between positive and negative classes in data sets and usually perform poorly on imbalanced data sets because it will maximize the overall prediction accuracy by a bias toward the majority class (52,54,55). Therefore, it will misclassify the minority class, in our case, which is the class of interest. To reduce the risk of the model performing poorly on the minority class (pre-miRNA), we solved the class imbalance problem at both data and algorithm levels by combining the SMOTE over-sampling method with the under-sampling method, and integrating them into the ensemble model. Various resampling methods have their own strengths and drawbacks. It was previously reported that under-sampling the majority class potentially removes certain important samples, resulting in loss of useful information. On the other hand, randomly over-sampling the minority class can lead to over-fitting on multiple copies of minority class examples (50,52,54). To avoid the problem of over-fitting, the technique called SMOTE was utilized to generate synthetic examples along the line segments joining any of the k minority class to their nearest neighbors; this broadened the decision boundaries for the minority class to spread further into the majority class space. At the algorithm level, our model is an ensemble of classifiers, one way to deal with the data imbalanced problem. Comparison of the effectiveness of several ensemble-based techniques in learning from imbalanced noisy data has shown that bagging techniques generally outperform boosting in most cases—bagging improved over individual classifiers is more consistent on various data sets than boosting (30,56). Moreover, the positive synergy between resampling techniques and bagging algorithms has been observed when comparing various ensemble-based rebalancing techniques. The hybrid approaches of SMOTE and under-sampling in the bagging-based algorithm, called SMOTEbagging, outperformed others (57). The technique is similar to our imbalance-tackle method, except the SMOTE resampling rate. We set the SMOTE resampling rate at the constant rate of 50% (the synthetic data were generated for 50% of the original data in the minority class) to reduce computational time and the amount of synthetic samples that could possibly degrade the performance of classifiers. Using modified-SMOTEbagging, we combined the strength of the individual methods while lessening the drawbacks. The SMOTE method also increased the performance of ensembles by establishing diversity, one factor necessary in building efficient ensembles. Comparing Vote2 (imbalanced) and Vote3 (balanced), the sensitivity of Vote3 increased by 10% (from 85.1 to 94.8%), which is significantly higher than that of Vote2, whereas the specificity of Vote3 is slightly decreased (<1%) from that of imbalanced class data.

By applying rebalancing techniques to handle the imbalanced-class in the training data, we significantly improved the performance of our ensemble from 95.81 to 96.54% in terms of accuracy, and from 0.976 to 0.996 in terms of AUC. Vote3 ensemble model with selected features and trained on class-balanced data yielded the highest accuracy and balance between sensitivity and specificity value by the voting of 12 diverse and accurate classifiers. The results suggest that obtaining discriminatory features by the FS method and rebalancing data distribution by resampling method are essential pre-processing steps for yielding accurate prediction. Thus, the model Vote3 would be further used in comparing the performance to other existing methods.

### Comparison of predictive performance of our ensemble with other methods

We compared the performance of our ensemble algorithm with those of the other existing methods (8–9,12,13), each of which has published results testing on the same data available to download (the 1st testing data set). The results of the comparison with existing methods are given in Table 6. Our ensemble outperformed other methods on three data sets: *TE-H, IE-NC and IE-M*. For the *IE-NH*, the miPred was slightly better than our method. However, miPred gave the lowest performance in term of specificity or it did not perform well in filtering out the negative testing data (the *IE-NC and IE-M*). Specificity is the performance that the method can identify and filter for the negative class. The specificity and FPR are correlated: when the method has high specificity, the FPR will be lower (%FDR = 100 − %Sp). Our method efficiently lowered false positives with an FPR of 16.7% compared with other methods with the FPR between 17.25–31.32% in *IE-NC* testing data.

**Table 6.** The prediction performance of the automated classifier, yasMir, miPred, tripletSVM and our method evaluated the same testing data set

Test sets	Automated classifier (11)	yasMiR (10)	miPred (7)	Triplet SVM (6)	Our method
TE-H	94.30	93.77	93.50	87.96	**98.37**
IE-NH	94.91	94.11	**95.64**	86.15	95.31
					
IE-NC	77.71	82.95	68.68	78.37	**83.22**
IE-M	96.77	**100**	87.09	0	**100**

TE-H (123 human pre-miRNA and 246 pseudo hairpins), IE-NH (1918 pre-miRNA across 40 non-human species and 3836 pseudo hairpins), IE-NC (12 387 functional ncRNAs) and IE-M (31 mRNAs). The values are percentages of correct prediction for each method on each data set. The highest values are in bold.

We also used the *TE-CS* data set (as reported in 8,12,15) for comparison—this was composed of 581 pre-miRNAs. This data set allows us to evaluate and compare the sensitivity of our method with Triplet-SVM, yasMir and PmirP, trained on human miRNA hairpin data. As shown in Supplementary Table S3, among the human miRNA hairpin-trained method, our method had the highest accuracy (98.1%) when compared with the other four methods. yasMir was the second best with sensitivity of 95.3% followed by PmirP, mirExplorer and Triplet-SVM with accuracy of 94.0, 92.4 and 90.9%, respectively.

In order to compare various ML techniques, we used the ‘Common Test’ data set from mirExplorer (58) which allowed our method to compare with SVM, RF and boosting-based algorithms. As shown in Table 7, our bagging based algorithm performed better in both sensitivity and specificity value than SVM and RF algorithm based method. Both sensitivity and specificity of our method is comparable to mirExplorer, a boosting based method. However, our ensemble performed the best in identifying the 437 multi-loop pre-miRNAs. Moreover, the performances of our method and MirExplorer in classifying across species miRNA, 16 species ranging from animals to virus, were reported in Supplementary Table S4.

**Table 7.** Comparison of our method to other method on ‘Common test’ testing data set of mirExplorer

Method	Balance method	CV	SE	SP	Gm	ACC(%) Multiloop
Triplet-SVM (SVM)	–	–	88.40	83.50	0.859	N/A
MiPred (random forest)	–	–	84.34	93.56	0.888	N/A
microPred (SVM)	SMOTE	outer 5cv	90.50	66.43	0.775	54.23
mirExplorer (AdaBoost)	SMOTE + undersampling	outer 10cv	94.32	97.11	0.957	92.68
Our method (ensemble)	Modified-SMOTEBagging	10x5cv	95.11	97.91	0.965	97.25

SE, SP and ACC represent sensitivity, specificity and accuracy, respectively.

Besides, it has been known that the plant pre-miRNA is different from animal pre-miRNA in several aspects, mainly in hairpin loop structure and size, with size ranging from 60 to 500 nt and containing short loops and long stems. In order to compare our ensemble sensitivity with other existing methods trained on plants pre-miRNAs, we used the same testing data as PlantMiRNAPred. The comparison of our classifier performance with the results reported in (14) is given in Table 8. As many plant pre-miRNAs contain multi-loop (14), our method can classify them correctly with the highest accuracy. This can be inferred that our method is sensitive enough to identify pre-miRNAs with multi-loop. These results suggested that our method performs with the highest sensitivity across plant and animal species, followed by the yasMir (12), which is the second best when the 1st and 3rd testing data were tested. As a consequence, the yasMir method was also included in comparison in the next sections, in which we downloaded the yasMir program and performed the test on our 2nd and 4th testing data.

**Table 8.** Sensitivity performance on plant specie pre-miRNAs

Species	No. of sequences	Accuracy (%)				Our method
		PlantMiRNA Pred (12)	Triplet-SVM (6)	microPred (9)	yasMir (10)	
ath	180	92.22	76.06	89.44	97.78	99.44
osa	397	94.21	75.54	90.43	96.72	100
ptc	233	91.85	75.21	84.98	93.99	96.99
ppt	211	92.42	71.49	89.57	98.10	98.57
mtr	106	100	80.18	95.28	100	100
sbi	131	98.47	69.51	94.66	95.42	100
zma	97	97.94	66.97	93.81	97.94	96.90
gma	83	98.31	74.12	86.75	96.38	98.79
updated aly	191	97.91	70.98	91.62	100	100
updated gma	118	98.31	79.66	93.22	100	100

All methods were tested on the testing data set of PlantMiRNAPred (14).

### High sensitivity of our ensemble

We evaluated the predictive power of the ensemble by applying it to predict all known pre-miRNA taken from miRBase version 17 and up-to-date version 18 (the 2nd testing set). This testing data is an across species pre-miRNA containing all pre-miRNAs from animal, plant and virus species. Our ensemble can achieve high accuracy of 92.89, 97.38 and 94.17% when testing across 93 animal species, 52 plant species and 23 virus species, respectively (Supplementary Table S5). Our methods—trained using human, monocot plant and dicot plant species—is applicable to animal, plant and virus species with high accuracy. Although the miRBase is a main miRNA repository, it contains published pre-miRNAs from both experimental and computational results. In order to test solely on experimentally verified pre-miRNA, we retrieved pre-miRNAs from the miRNAMap (59). The testing results on pre-miRNAs from miRNAMap are given in Supplementary Table S6. Our method achieved high accuracy of 97.29% when testing on all experimentally verified pre-miRNA sequences from miRNAMap.

We compared the performance of our ensemble with its individual classifiers of SVM, kNN and RF; the results are shown in Supplementary Figure S3. We also included another existing method called yasMir (12), which is a SVM-based classifier, in the plot. As depicted in the plot, the ensemble got better prediction results compared with single SVM, kNN, RF and yasMir in most testing cases. The ensemble model is a high-performance approach, relatively, providing superior accuracy—higher than single classifiers. This is due to the complementary role from each of the 12 classifier members in our ensemble model. This result is consistent with the previous findings (30,56) that the bagging-based classifier is almost always more accurate than single individual classifiers in most testing cases while the boosting-based classifier could be less accurate than single individual classifiers in some cases.

Not only does the algorithm affects performance of prediction, but also our discriminative features, SC and its derivatives, to improve the efficiency of our model. To give the supported evidences that our novel features would significantly distinguish real pre-miRNAs from other stem-loop sequences in the testing data, average values of SC and its SC-base pair composite features, across different groups of organisms in our testing data, including those of negative data set are calculated and presented in Supplementary Table S7. Average values of MFE, a well-known feature, across different groups of organisms were also given. The MFE values of small ncRNAs fall into the range of −33.16 ± 24.17 similar to those of animal pre-miRNAs. In addition, we observed high MFE and high variation in MFE distribution of plant pre-miRNAs. This shows that MFE can be used to distinguish pre-miRNAs from random pseudo hairpins, but cannot differentiate the real pre-miRNAs from other small stem-loop forming ncRNAs. Consistent with the training data, the average values of SC and three SC-base pair composite features of all pre-miRNAs in testing data were significantly higher than those of other negative hairpin sequences. The distributions of MFE, SC and SC derivative values for the testing data were plotted as shown in Supplementary Figure S4. In contrast to the MFE, well separations between positive and negative data were found in SC and SC-base pair composite features. The SC and our three SC-base pair composite features are useful for distinguishing real pre-miRNA hairpins, both plant and animal, from pseudo hairpin and other ncRNA sequences effectively. Moreover, the viral pre-miRNAs, known to evolve rapidly from plant and animal pre-miRNAs (60) also show a similar trend in SC and SC-base pair composite features to plant and animal pre-miRNAs. This confirmed that the pre-miRNAs possess unique functional structure that distinguishes them from other hairpin structures.

### High specificity of our ensemble

The ability to reduce FPR is essential in the computational identification of pre-miRNA sequences. To assess the FPR of our ensemble, we compared our method with yasMir, the second best performance in terms of sensitivity, on the 4th testing data set. The results showed that the ensemble had the FPR of 6.26% for classifying miRNA from pseudo-hairpins, 11.65% for classifying miRNA from shuffle sequences, and 16.78% for classifying miRNA from other functional ncRNA (Table 9). This suggested that the method had a low FPR (11.56%), which was relatively low for scanning pre-miRNA sequences in genomes compared with the yasMir algorithm. We also applied our method in a more realistic situation as a computational pipeline for pre-miRNA scanning on the genome scale as reported in Supplementary Method S5. The ensemble, the voting of multi-expert classifiers, is known as an effective way of increasing specificity through voting and of giving lower false positive results than a single classifier. Our *ab-initio* ensemble based method has proved in this and previous sections that it can predict pre-miRNAs with high sensitivity and specificity.

**Table 9.** Specificity of our ensemble when applied to the negative testing data, compared with yasMir (the 2nd best sensitivity from Table 8)

Negative data	No. of sequences	Our method		yasMir	
		Correctly classified (%)	FPR (%)	Correctly classified (%)	FPR (%)
Pseudo hairpin	4494	93.74	6.26	86.91	13.09
Shuffle	21 470	88.35	11.65	83.69	16.31
IE-NC (1238ncRNA)	12 387	83.22	16.78	82.95	17.05
Average	12 784	88.44	11.56	84.52	15.48

Correctly classified (%) is the percent of the correctly classified as not pre-miRNAs, FPR (%) is the false positive rate.

The accuracy of the method can be affected by the reliability of the training data. A recent study (61) demonstrated that commonly used positive and negative control data may be unreliable, and provided a new set of control data: high confidence positive control data with functional evidences and negative control data with no evidence of processing by Dicer. Our method was also tested with these novel control data. It yielded accuracy of 100% for positive control and accuracy of 98.09% for negative data. As given in Supplementary Method S6, our method predicted almost all positive control (127 out of 129) as pre-miRNA with the highest probability of 1.0, whereas 2 of 129 were predicted as pre-miRNA with high probability of 0.75. This result again confirmed that our discriminative features and algorithm work well in identifying bona fide functional pre-miRNAs.

## CONCLUSION

Various ML algorithms—including NB, MLP, J48, SVM, kNN, RBFNets, RIPPER and RF—were applied to discriminate real microRNA precursors from pseudo-hairpin sequences and other ncRNAs. The comparison performance of each algorithm on the pre-miRNA classification task was performed. Since different learning algorithms have different strengths and weaknesses, we proposed to apply a heterogeneous ensemble to improve miRNA hairpin classification. The heterogeneous ensemble method has shown to improve the performance in terms of sensitivity-specificity tradeoff. The method contributes towards an improvement of miRNA hairpin classification by the following reasons. Firstly, this vote of multiple diverse classifiers could have better and more reliable prediction than a single classifier since it can reduce the chance of incorrect classification in single algorithms. Secondly, the ensemble incorporated with the modified-SMOTEbagging techniques is an effective way to handle class-imbalanced problems occurring in pre-miRNA data. Each base classifier in the ensemble is trained on a well-balanced subset of the training data, which makes our model better for classifying the minority class (pre-miRNAs) than those of class-imbalanced data. Thirdly, the ensemble can give an optimized answer with respect to sensitivity, specificity and accuracy by selected RF (one member that gives the highest performance in identifying the positive class), selected kNN (one member that gives the highest performance in filtering out the negative class) and selected SVM (one algorithm in the ensemble that can give better tradeoff between true positive and false positive), respectively. The aggregation of these algorithms increased the possibility that the ensemble truly represented the characteristics of pre-miRNAs. Finally, our ensemble also incorporated robust features, that is, our SC-base pair composite features, proven to be the most informative from the feature set that can efficiently discriminate true pre-miRNA hairpins.

Unlike previous methods, ours was trained on the data set containing human and plant pre-miRNAs. The overall CV prediction accuracy was 96.54% for our ensemble, which significantly outperformed all other learning methods at 95% confidence level. We also tested the performance of the ensemble on cross-species data taken from miRBase18. The results demonstrated that the method performs well across animal, plant and virus species with accuracy of 92.89, 97.38 and 94.17%, respectively. In conclusion, integrating the resampling techniques and discriminative feature set to the miRNA heterogeneous ensemble classification algorithm can improve the accuracy of miRNA hairpin classification.

## SUPPLEMENTARY DATA

Supplementary Data are available at NAR Online: Supplementary Tables 1–7, Supplementary Figures 1–4, Supplementary Methods 1–6, Supplementary Data 1–2 and Supplementary References [62–73].

## FUNDING

National Research University Project of Thailand’s Office of the Higher Education Commission [54000318]. Funding for open access charge: King Mongkut's University of Technology Thonburi.

*Conflict of interest statement*. None declared.

## Supplementary Material

Supplementary Data
