# Supplementary material for: Heterogeneous ensemble approach with discriminative features and modified-SMOTEbagging for pre-miRNA classification
Source: Nucleic Acids Res. 2012 Sep 24;41(1):e21. doi: 10.1093/nar/gks878 (PMC3592496; doi:10.1093/nar/gks878)
Supplement: Supplementary Data [file supp_41_1_e21__index.html]

Heterogeneous ensemble approach with discriminative features and modified-SMOTEbagging for pre-miRNA classification — Supplementary Data 

# Heterogeneous ensemble approach with discriminative features and modified-SMOTEbagging for pre-miRNA classification

## Supplementary Data

files

**Files in this Data Supplement:**

- Supplementary Data - xlsx file
- Supplementary Data - zip file
- Supplementary Data - docx file
