# Supplementary material for: Heterogeneous ensemble approach with discriminative features and modified-SMOTEbagging for pre-miRNA classification
Source: Nucleic Acids Res. 2012 Sep 24;41(1):e21. doi: 10.1093/nar/gks878 (PMC3592496; doi:10.1093/nar/gks878)
Supplement: Supplementary Data [file supp_gks878_Revised_SupplementaryFile_gks878.docx]

# Supplementary Materials and Methods for:

Heterogeneous Ensemble Approach with Discriminative Features and Modified-SMOTEbagging for Pre-miRNA Classification

Supatcha Lertampaiporn^1^, Chinae Thammarongtham^2^, Chakarida Nukoolkit^3^, Boonserm Kaewkamnerdpong^1^, Marasri Ruengjitchatchawalya^4,5^*

^1^Biological Engineering Program, King Mongkut’s University of Technology Thonburi, Bang Mod, Thung Khru, Bangkok, 10140, Thailand

^2^Biochemical Engineering and Pilot Plant Research and Development Unit, National Center for Genetic Engineering and Biotechnology at King Mongkut’s University of Technology Thonburi, Bang Khun Thian, Bangkok, 10150, Thailand

^3^School of Information Technology, King Mongkut’s University of Technology Thonburi, Bang Mod, Thung Khru, Bangkok, 10140, Thailand

^4^School of Bioresources and Technology, King Mongkut’s University of Technology Thonburi, Bang Khun Thian, Bangkok, 10150, Thailand

^5^Bioinformatics and Systems Biology Program, King Mongkut’s University of Technology Thonburi, Bang Khun Thian, Bangkok, 10150, Thailand

* To whom correspondence should be addressed. Tel: +66-2-470-7481; Fax: +66-2-452-3455; E-mail: marasri.rue@kmutt.ac.th

# Supplementary Materials and Methods 1

The 4 testing sets used in this work:

The 1^st^ testing dataset consists of various testing dataset of the miPred (9), triplet-SVM (8), and MirExplorer (58) websites. The testing data is composed of *TE-H* (containing 123 human pre-miRNAs and 246 randomly chosen pseudo hairpins)*, IE-NH* (containing 1,918 pre-miRNAs across 40 non-human species and 3,836 randomly selected negative examples)*, IE-NC* (containing 12,387 function ncRNA from Rfam 7.0), *IE-M* (containing 31 mRNAs from the genBank DNA database)*, TE-CS* (containing 581 pre-miRNAs from 11 different species from miRBase version5) and two datasets from miRExplorer (58); the “*Common Test*” (containing 862 real and 12,930 pseudo pre-miRNAs) and the “*CROSS-SPECIES*” (containing 1993 pre-miRNAs of 16 species from miRBase version 12)*.* These dataset have been used in publications (Triplet-SVM (8), miPred (9), microPred (11), yasMir (12), automated classifier (13), plantMiRNAPred (14), PmirP (15), and MirExplorer (58)), allowing us to evaluate and compare the performance of our method with other methods.

The 2^nd^ testing dataset was used as a testing set to investigate performance of our proposed model on cross-species pre-miRNAs. This testing data set obtained from a main miRNA repository database, the miRBase database version 17 and 18 (up-to-date version of miRBase at the time of this manuscript). However, the well-known miRBase contains already published results based on both computational and experimental methods, other database that exclusively consists of experimentally verified miRNA was necessary in testing the algorithm. In addition to pre-miRNA data from miRBase, we also obtained all 12 species of pre-miRNA from miRNAMap version2 (59).

The 3^rd^ testing dataset was the PlantMiRNAPred testing dataset (14). This testing dataset was composed of 180 pre-miRNAs of *A. thaliana*, 397 of *O. sativa*, 233 of *Populus trichocarpa*, 211 of *Physcomitrella patens*, 106 of *Medicago truncatula*, 131 of *Sorghum bicolor*, 97 of *Zea may*s, 83 of *Glycine max*, 191 of updated *A. lyrata* , and 118 of updated *G. max*.

The 4^th^ testing dataset was aimed to assess the specificity of our method. It was composed of 3 different negative data as follows: the remaining 4,494 from 8,494 pseudo hairpin sequences (after selecting the 4,000 hairpins as a training data), 21,470 shuffled pre-miRNA sequences, and the IE-NC from the 1^st^ testing dataset. The 21,470 shuffled sequences are generated from each of the 2,147 real pre-miRNA sequences (491 of *O. sativa*, 1,424 of *H. sapiens*, 232 of *A. thaliana* from miRBase17) shuffling 10 times by (a) 5 times generating 5 random shuffles preserving the same mononucleotide base composition and (b) 5 times generating 5 random shuffles preserving mono- and di-nucleotide base composition. This testing dataset was used as negative testing data to investigate the false positive rate and specificity of our proposed model. The data are summarized as the diagram in Figure S1.


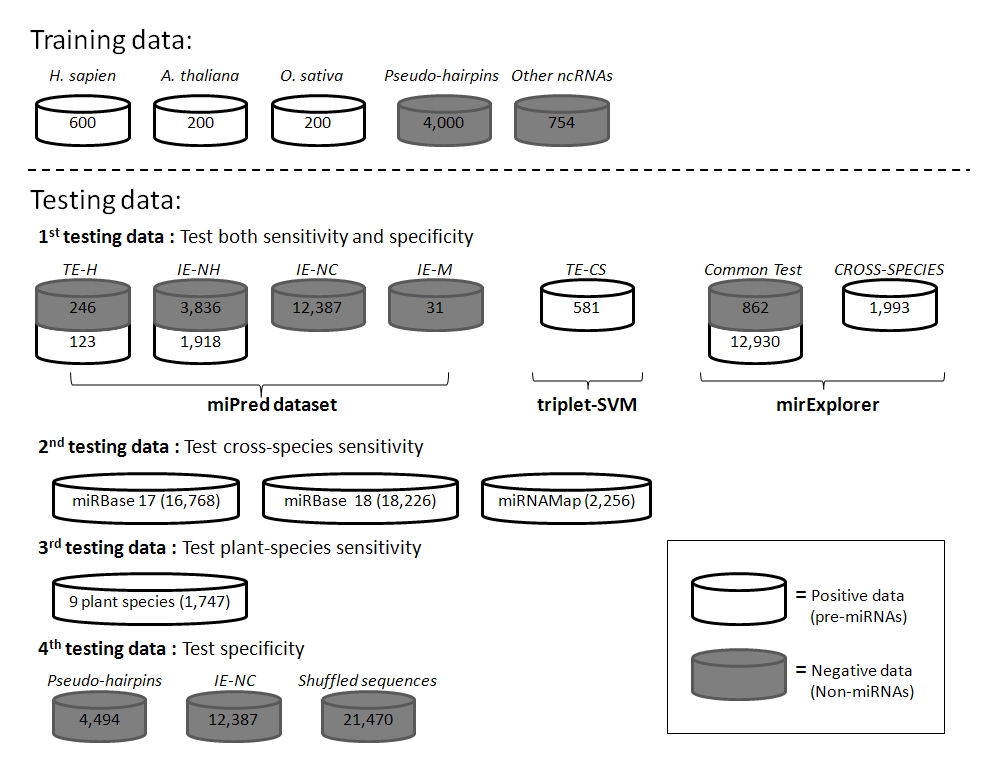


# Figure S1 Various datasets used in this work. The negative and positive data are shown with different colors: The training data consist of three species pre-miRNA as positive data and two type of non-miRNA as negative data. We used four testing datasets for testing the performance. The 1^st^ testing used to compare both sensitivity and specificity value with other previous proposed methods. The 2^nd^ testing data is across specie pre-miRNAs from miRBase and miRNAMap databases. The 3^rd^ testing data is composed of plant species pre-miRNAs which allow our method to compare the performance with others which were applied to plant species. Lastly, the 4^th^ testing data includes three types of negative data (non-miRNAs) that we used to test the specificity (and false positive rate) of our method.

# Supplementary Materials and Methods 2

**Features**

In order to discriminate miRNA hairpins from other hairpins, it is necessary to find features characterizing the miRNA hairpin. The features in this study fall into five distinct properties: primary sequence based property, secondary structure property, base-pair distance property, triplet sequence-structure property, and structural robustness features.

*Primary sequences based features*

These sequence-based features consist of G+C% content, A+U% content, 16 di-nucleotide frequencies (%AA, %AC, %AG, %AU, %CA, %CC, %CG, %CU, %GA, %GC, %GG, %GU, %UA, %UC, %UG, %UU), and sequence length (Len).

*Secondary structure and thermodynamic stability features*

Secondary structure features are based on thermodynamics represented by free energy minimization (Zuker algorithm; Mfold (62)) and partition function method (Mccaskill algorithm; RNAfold (63-66)). These folding features included MFE (minimum free energy), MFEI1 (MFE index1) (67), MFEI2 (MFE index2), MFEI3 (MFE index3), MFEI4 (MFE index4), dG, dQ, dD, dF, zG, zQ, zD, zF, Prob, Freq, diff, efe, nefe, dH, dH/L, dS, dS/L, Tm, Tm/L.

dG is normalized minimum free energy per length (MFE/Length), MFEI1 is ratio between dG and %G+C (= dG/%G+C)(68), MFEI2 is ratio dG and the number of Stem (= dG/Stem), MFEI3 is ratio of dG and the number of loops in the secondary structure, MFEI4 is ratio of minimum free energy and total number of base pairs in secondary structure (=MFE/tot_bp), efe is ensemble free energy, nefe is normalized ensemble free energy (=efe/Length), freq is frequency of the MFE structure, diff is different between MFE and efe (=|MFE-efe| / length), Q is Shannon entropy, dQ is normalized Q by length (Q/Length), D is average base-pair distance, dD is normalized D by length (D/Length), zX or z score of X is number of standard deviation by which x differ from the mean of all shuffled (x) sequences having the same di-nucleotide composition. Prob is probability that the MFE of the given RNA sequence is different from a distribution of MFE computed with random sequences (68). The detailed calculation of Z score and Prob value is described in (68-69). dF is compactness degree of tree-graph representation of the sequence.

*Base-pair distance features*

The base-pair features calculated included dP, zP, Div, tot_bp, stem, loop, A-U/L, %A-U/Stem, G-C/L, %G-C/Stem, G-U/L, %G-U/Stem, Probpair1-10, Avg_BP_stem, NonBP_A, NonBP_C, NonBP_G, NonBP_U, Non_BPP.

Where tot_bp is total number of base pairs in the secondary structure, dP is normalized base-pairing propensity (= tot_bp/Length), zP (Z score of dP) is number of standard deviation by which dP differ from the mean of all shuffled (dP) sequences having the same dinucleotide composition, Div is the structural base-pair distance, X-Y is number of base X paired with base Y in the secondary structure, %X-Y is percentage of base X paired with base Y in the secondary structure, Loop is number of loops in the structures, and Stem is number of stems in the structures, which are structural motifs containing more than three contiguous base pairs. Probpair is sum of pairing probabilities for each pair of nucleotides (AA, AC, AG, AU, CC, CG, CU, GG, GU, UU). Avg_BP_stem is ratio of number of base-pairing and the stem. NonBP_A is non-pairing probability for nucleotide A. NonBP_C is non-pairing probability for nucleotide C. NonBP_G is non-pairing probability for nucleotide G. NonBP_U is non-pairing probability for nucleotide U. Non_BPP is overall non base-pairing probability.

*Triplet structure-sequence Features*

The features focus on the information of every 3 adjacent nucleotides that combined the local contiguous structures with sequence information to characterize the pre-miRNA structure. For any 3 adjacent nucleotides, there are 8 possible structures: “(((“, “((**.**”, “(**..**”, “(**.**(“, “**.**((“, “**.**(**.**”, “**..**(“and “**…**” where brackets “(“ represented paired nucleotide and dot “.” represented unpaired nucleotide. Considering the middle nucleotide among the adjacent nucleotides which can be A, C, G, or U base, then there are 32 possible structure-sequence combinations. More detail is described (8).

*Structural robustness features*

In order to improve pre-miRNAs classification, we take into account more auxiliary information. Other than static thermodynamic folding properties (MFE), other features may be needed for effective finding of miRNA hairpin due to the fact that many random genomic background sequences can form stem-loop hairpin with high MFE value.

We incorporated structural robustness related properties into our feature set. The self-containment index score (SC) showed that predicted pre-miRNA results exhibit an enhanced degree of SC characteristic between 0.85 and 0.98 because of the need of pre-miRNA to maintain structural invariance through cleavages step during its biogenesis. Because of this invariant structure through the biogenesis of pre-miRNA, the pre-miRNA stem loops exhibit high self containment, while pseudo miRNAs and other structured RNA do not (39). The SC is calculated as followed: for each RNA sequence w of length L folding into a particular minimum free energy secondary structure, the original sequence then embedded in between randomly generated sequence x and y of equal length, forming a concatenated molecule xwy of length 3L. The embedded larger sequence is folded and measured the proportion of the original structure preserved in the larger structure. This process repeat 1000 times to generate a single statistic value ranging from 0.0 to 1.0, with 1.0 indicating a maximal degree of self containment. The miRNA has ability to remain robust over many different genomic contexts with SC scores between 0.86 and 0.98 reflecting its structural invariance during its biogenesis mechanism.

To increase the sensitivity of the SC score, we introduced novel features called SC derivative features which are incorporated other information of RNA hairpin structure, such as dP, (1-dP), tot_bp, NonBP_A, NonBP_C, NonBP_G, NonBP_U, Len, MFE, Mean_dG and zG into the SC score.

Where SCxzG is a product of feature SC and a zG, SCxdP is a product of SC and dP, SC/(1-dP) is a normalized SC by (1-dP) where dP is proportion of base-pairing in structure (=tot_bp/Length = dP), (1-dP) is proportion of non-pairing nucleotides in structure.

*Additional derivatives of features*

We also included new additional features which are 10 derivative features such as MFEI5, MFE/Mean_dG, dH/loop, dS/loop, Tm/Loop, dQ/loop, Avg_BP_Loop, %A-U/BP, %G-C/BP and %G-U/BP into our feature set. Where Loop is number of loops in the structure, BP is total number of base-pair in structure, MFEI5 is ratio of minimum free energy and the CG content in the base pairs (=MFExCG/tot_bp), Mean_dG is mean of all shuffled sequences having the same dinucleotide composition.

# To assess the performance of each feature group, a single SVM classifier was trained using LIBSVM (47) with default parameters (RBF kernel, c=1, γ =1/no. of feature). The SVM was used because it is fast and easy to implement.

# Supplementary Materials and Methods 3

Since ReliefF and InfoGain methods determine the relevance of each feature to the target class by ranking based methods, we need to find the optimal number of feature for each criterion to build a classifier. To determine the optimal number of feature for InfoGain and ReliefF ranking method, the accuracy performance versus number of the top *n* discriminating features was plotted. We considered the top ranking *n* features by n ranged from 5, 10, 15, 20, 25, 30, 35, 40, 45, 50, 55, 60, 65, 70, 75, 80, 85, 90, 95, 100, 105, 110, 115, 120, and 125 features. With the given criterion, the result of the 3-fold cross validation accuracy using the *n* top raking features are plotted as shown in the Figure S2. The InfoGain method achieved its highest performance when using its top 75 ranking features while the ReliefF method achieved its highest performance when using its top 50 ranking features.


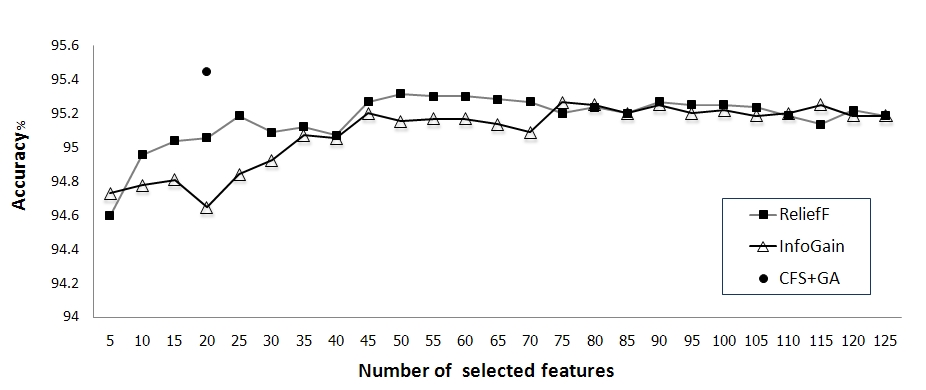


**Figure S2.** Average 3-fold CV performance when using the n top ranked features of ReliefF and infoGain methods. To determine the optimal number of feature for *Relief*F and *InfoGain*, we plotted the average 3-fold CV accuracy when using *n* top ranked feature. While the CFS method selects the optimal feature subset by the search method, so plotting the graph to find the optimal number of feature is not necessary. The “*black dot*” representing the average 3-fold cross validation accuracy of the *CFS+GA* features subset.

To find the optimal feature subset by the CFS method, genetic algorithm (GA) was used as a search method. The merit score of CFS is used as a fitness function in GA search. The parameter setting for our implementation of GA search included cross-over probability of 0.8, a mutation probability of 0.05, and a population size of 200. The GA search started with the initial feature set of 20 top ranked features from ReliefF criterion and the GA search terminated when the merit score (fitness function) converged or reached the maximum 1,000 generation.

For feature selection method assessment, SVM with default LIBSVM (47) parameters (c=1, γ=1/no. of feature) was used and compared to the 3-fold CV generalization accuracy of the SVM from the various feature selection subsets.

Interestingly, using different feature selection methods resulted in different feature ranking, however, our new introductory feature, i.e. SC-Base Pair composite features -SCx%BP, SC/%NonBP, SCxBP/NonBP, were always in the top 5 ranking, implying our SC-Base pair composite features quite robust features. We also included the top 15 ranking features by Relief, InfoGain, and OneR criteria in the Table S1.

**Table S1.** The top 15 ranked individual features by Relief, InfoGain, and OneR

| **Ranking** | **Information Gain Ranking** | **ReliefF Ranking** | **OneR Ranking** |
| --- | --- | --- | --- |
| 1  2  3  4  5  6  7  8  9  10  11  12  13  14  15 | MFEI1  SCxdP/(1-dP)  SCxdP  SC/(1-dP)  SCxzG  zG  SCxMFE/Mean_dG  SC/NonBP_A  SC  Non_BPP  Prob  dP  zQ  zP  Avg_BP_Loop | SC  Prob  SCxdP  SC/(1-dP)  SCxdP/(1-dP)  Non_BPP  A-U/L  dP  %A-U/BP  NonBP_A  SCxMFE/Mean_dG  NonBP_C  dG  nefe  Tm | SCxdP/(1-dP)  MFEI1  SC/(1-dP)  SCxdP  SCxzG  zG  Prob  SCxMFE/Mean_dG  SC  SC/NonBP_A  Non_BPP  Avg_BP_Loop  zP  SC/NonBP_U  MFEI5 |

# Supplementary Materials and Methods 4

# Eight different classification algorithms were obtained from WEKA Machine Learning Library (46) with default parameter settings unless otherwise stated.

1. Naïve Bayes (NB)
2. Neural networks using multi layer perceptron (MLP) (with default parameters for MLP in WEKA: number of epochs: 500; learning rate: 0.3; and momentum of updating weights: 0.2)
3. Support vector machine (SVM). 2 SVM models were considered: 1 SVM with polynomial kernel in WEKA and 1 SVM with RBF (radial bias function) kernel using LIBSVM (47,48). To explore a wide range of parameters, c and γ, we used the grid.py script to search for optimal c and gamma value for the RBF kernel: c=128 and γ =0.125. Comparing these 2 SVM models, the SVM with RBF kernel outperformed the SVM with polynomial kernel. Thus, the LIBSVM with RBF was used further as a benchmark.
4. K-nearest neighbor (kNN): 21 kNN with the combination of k =1, 3, 5, 7, 9, 12, and 15 and 3 types of weighting method: no weight, inverse weight, and similarity weight were considered. We used a MultiScheme package in WEKA to choose between 21 kNN models and the kNN with k=9 and inverse weighting yielded the best result. Thus, the kNN with k=9 and inverse weighting was used further as a benchmark.
5. Decision Tree (J48) is an implementation of C4.5.
6. Repeated Incremental Pruning to Produce Error Reduction (RIPPER) is rule induction based learning. We have implemented this rule based system in WEKA using JRIP with default parameters.
7. RBF Networks (RBFNets).
8. Random forest (RF) is a homogeneous ensemble of decision tree. The number of trees was 10 and the number of features was 5.

# Supplementary Materials and Methods 5

**Computational pipeline for genome scale (genome-wide) prediction of miRNA precursors**

In this section, we demonstrate the effectiveness of our approach as a computational pipeline for pre-miRNA prediction in genome-wide scale. We used 2 types of sequences which have been used by Tempel et al (70). These sequences were available for downloading at **Supplementary data 2**.

1) Artificial genomic sequence is the concatenation of human mRNA and the insertion of 100 human pre-miRNAs. The human mRNA sequences were obtained from the Human genome of NCBI Website and the pre-miRNAs were retrieved from the miRBase database.

2) Real genomic sequences from human genome chromosome 19 (strand +) has a cluster of 50 pre-miRNAs as described in Tempel et al (70). The sub-sequence that includes the considered pre-miRNAs cluster was extracted from NCBI website and given in **Supplementary data 2**.

Our Perl-based pipeline for large-scale miRNA precursor prediction consists of 2 main components 1) a sliding-window module for scanning through the genome sequence 2) module containing a classification model for scoring each window based on ensemble of various classifiers. The pipeline starts from generating 100-nt sequences from the genome sequence by sliding window of size 100 nt along the genome sequence. Then each fragmented sequence was scored by our classification model as a pre-miRNA or other. The classification result also provides the probability of the voting prediction which indicates the reliability of each prediction (we used the probability > 0.75 for predicting sequence as a reliable pre-miRNA).

**TableS2.** Result obtained on an artificial sequence and a Human Genome chr19

| **Method** | **Artificial Sequence** | | **Real Human Sequence** | | **Ref.** |  |
| --- | --- | --- | --- | --- | --- | --- |
|  | **Sensitivity** | **Specificity** | **Sensitivity** | **Specificity** |  |  |
| CID-miRNA  miRPara  VMir  miRNAFold_50-80_  miRNAFold_90_  Our Method | 97  97  28  96-98  65  97 | 11.72  9.7  1.32  18.77-22.91  52  72.38 | 38  98  100  100  N/A  100 | 0.69  0.93  0.56  0.89  N/A  0.91 | 71  72  73  70  70 |  |

We reported our genome search results in **Table S2** and compared with CID-miRNA, miRPara, VMir and miRNAFold (70-73). By scanning the artificial genome sequence, our method exhibited high sensitivity in identifying 97 out of 100 real miRNA precursors and high specificity of 72.38%. This means that the method would detect 97 of 100 pre-miRNAs while producing a low false positive rate. In our result, three real pre-miRNAs were missed out of the prediction. It was found that these three sequences were scored with the voting probability of 0.5 which is lower than our cutoff value. Therefore they were not reported in the result since we set the stringent threshold at 0.75 to identify the sequence as pre-miRNA. However, the voting probability also provides some hints about the pre-miRNA characteristic in which half of the classifiers in ensemble voted for these 3 sequences as pre-miRNA. This implies that when the sequence of interest does not have high pre-miRNA characteristic so the committees in the ensemble are not in consensus to vote the sequence of interest as pre-miRNA. The disagreement of committee is consequent from using modified-SMOTEbagging and heterogeneous ensemble techniques; each committee in ensemble is expert in different training space and different learning processes. When the diverse committees agreed on the consensus prediction means the prediction have the high reliability. In the future, we may improve the prediction performance of our method by giving the weighted voting for each classifier in ensemble and providing the detailed result to users by telling which classifier vote for what by which learning algorithm. By doing that would enable a user to get even more reliable prediction. By scanning the real genome data, our method is sensitive enough to identify all 50 known pre-miRNAs with the high specificity of 0.91% compared to others.

In comparison to the existing methods, our ensemble pipeline system achieved high accuracy in sensitivity and selectivity as well as betters in trade-off between these two values. In this section, we proved that our method efficient for analyzing genome-scale data. Comparing to the existing methods, our proposed ensemble system achieved high accuracy in sensitivity, specificity, and overall accuracy. Moreover, our prediction result provided the voting probability, voting of various prediction algorithms in ensemble, which can tell the user about the reliability of each prediction.

# Supplementary Materials and Methods 6

# === Predictions on “MMU-high confidence” test data ===

# inst# actual predicted pred.prob. (SC score)

# 1 1:miRNA 1:miRNA 1 (0.896392)

# 2 1:miRNA 1:miRNA 1 (0.942353)

# 3 1:miRNA 1:miRNA 1 (0.801912)

# 4 1:miRNA 1:miRNA 1 (0.978788)

# 5 1:miRNA 1:miRNA 1 (0.919429)

# 6 1:miRNA 1:miRNA 1 (0.984923)

# 7 1:miRNA 1:miRNA 1 (0.955357)

# 8 1:miRNA 1:miRNA 1 (0.951642)

# 9 1:miRNA 1:miRNA 1 (0.768506)

# 10 1:miRNA 1:miRNA 1 (0.958182)

# 11 1:miRNA 1:miRNA 1 (0.982237)

# 12 1:miRNA 1:miRNA 1 (0.993333)

# 13 1:miRNA 1:miRNA 1 (0.928904)

# 14 1:miRNA 1:miRNA 1 (0.93381)

# 15 1:miRNA 1:miRNA 1 (0.846881)

# 16 1:miRNA 1:miRNA 1 (0.389762)

# 17 1:miRNA 1:miRNA 1 (0.907595)

# 18 1:miRNA 1:miRNA 1 (0.964507)

# 19 1:miRNA 1:miRNA 1 (0.85225)

# 20 1:miRNA 1:miRNA 1 (0.8887)

# 21 1:miRNA 1:miRNA 1 (0.940952)

# 22 1:miRNA 1:miRNA 1 (0.987403)

# 23 1:miRNA 1:miRNA 1 (0.994138)

# 24 1:miRNA 1:miRNA 1 (0.84925)

# 25 1:miRNA 1:miRNA 1 (0.909077)

# 26 1:miRNA 1:miRNA 1 (0.98814)

# 27 1:miRNA 1:miRNA 1 (0.989296)

# 28 1:miRNA 1:miRNA 1 (0.919242)

# 29 1:miRNA 1:miRNA 1 (0.905424)

# 30 1:miRNA 1:miRNA 1 (0.756463)

# 31 1:miRNA 1:miRNA 1 (0.983235)

# 32 1:miRNA 1:miRNA 1 (0.914167)

# 33 1:miRNA 1:miRNA 1 (0.987009)

# 34 1:miRNA 1:miRNA 1 (0.939859)

# 35 1:miRNA 1:miRNA 1 (0.987857)

# 36 1:miRNA 1:miRNA 1 (0.909101)

# 37 1:miRNA 1:miRNA 1 (0.981452)

# 38 1:miRNA 1:miRNA 1 (0.940274)

# 39 1:miRNA 1:miRNA 1 (0.990278)

# 40 1:miRNA 1:miRNA 1 (0.978983)

# 41 1:miRNA 1:miRNA 1 (0.881705)

# 42 1:miRNA 1:miRNA 1 (0.992099)

# 43 1:miRNA 1:miRNA 1 (0.87197)

# 44 1:miRNA 1:miRNA 1 (0.987353)

# 45 1:miRNA 1:miRNA 1 (0.989178)

# 46 1:miRNA 1:miRNA 1 (0.984545)

# 47 1:miRNA 1:miRNA 1 (0.9275)

# 48 1:miRNA 1:miRNA 1 (0.961053)

# 49 1:miRNA 1:miRNA 1 (0.969873)

# 50 1:miRNA 1:miRNA 1 (0.912716)

# 51 1:miRNA 1:miRNA 1 (0.8)

# 52 1:miRNA 1:miRNA 1 (0.8544)

# 53 1:miRNA 1:miRNA 1 (0.948286)

# 54 1:miRNA 1:miRNA 1 (0.965362)

# 55 1:miRNA 1:miRNA 1 (0.986462)

# 56 1:miRNA 1:miRNA 1 (0.993803)

# 57 1:miRNA 1:miRNA 1 (0.921324)

# 58 1:miRNA 1:miRNA 1 (0.982973)

# 59 1:miRNA 1:miRNA 1 (0.866026)

# 60 1:miRNA 1:miRNA 1 (0.948587)

# 61 1:miRNA 1:miRNA 1 (0.8524)

# 62 1:miRNA 1:miRNA 1 (0.954048)

# 63 1:miRNA 1:miRNA 1 (0.791286)

# 64 1:miRNA 1:miRNA 1 (0.742308)

# 65 1:miRNA 1:miRNA 1 (0.794)

# 66 1:miRNA 1:miRNA 1 (0.961098)

# 67 1:miRNA 1:miRNA 1 (0.991585)

# 68 1:miRNA 1:miRNA 1 (0.922911)

# 69 1:miRNA 1:miRNA 1 (0.983173)

# 70 1:miRNA 1:miRNA 1 (0.884286)

# 71 1:miRNA 1:miRNA 1 (0.837397)

# 72 1:miRNA 1:miRNA 1 (0.931081)

# 73 1:miRNA 1:miRNA 1 (0.941829)

# 74 1:miRNA 1:miRNA 1 (0.929518)

# 75 1:miRNA 1:miRNA 1 (0.891047)

# 76 1:miRNA 1:miRNA 1 (0.979683)

# 77 1:miRNA 1:miRNA 1 (0.950494)

# 78 1:miRNA 1:miRNA 1 (0.897442)

# 79 1:miRNA 1:miRNA 1 (0.985465)

# 80 1:miRNA 1:miRNA 1 (0.95)

# 81 1:miRNA 1:miRNA 1 (0.955532)

# 82 1:miRNA 1:miRNA 1 (0.602989)

# 83 1:miRNA 1:miRNA 1 (0.91527)

# 84 1:miRNA 1:miRNA 1 (0.992111)

# 85 1:miRNA 1:miRNA 1 (0.788519)

# 86 1:miRNA 1:miRNA 1 (0.958989)

# 87 1:miRNA 1:miRNA 1 (0.843218)

# 88 1:miRNA 1:miRNA 1 (0.975854)

# 89 1:miRNA 1:miRNA 1 (0.901429)

# 90 1:miRNA 1:miRNA 1 (0.937955)

# 91 1:miRNA 1:miRNA 1 (0.875435)

# 92 1:miRNA 1:miRNA 1 (0.953415)

# 93 1:miRNA 1:miRNA 1 (0.946707)

# 94 1:miRNA 1:miRNA 1 (0.990682)

# 95 1:miRNA 1:miRNA 1 (0.895244)

# 96 1:miRNA 1:miRNA 1 (0.835862)

# 97 1:miRNA 1:miRNA 1 (0.966709)

# 98 1:miRNA 1:miRNA 1 (0.986962)

# 99 1:miRNA 1:miRNA 1 (0.970357)

# 100 1:miRNA 1:miRNA 1 (0.975606)

# 101 1:miRNA 1:miRNA 1 (0.939394)

# 102 1:miRNA 1:miRNA 1 (0.97697)

# 103 1:miRNA 1:miRNA 1 (0.985974)

# 104 1:miRNA 1:miRNA 1 (0.976582)

# 105 1:miRNA 1:miRNA 1 (0.955333)

# 106 1:miRNA 1:miRNA 1 (0.893039)

# 107 1:miRNA 1:miRNA 1 (0.909882)

# 108 1:miRNA 1:miRNA 1 (0.882222)

# 109 1:miRNA 1:miRNA 0.75 (0.681463)

# 110 1:miRNA 1:miRNA 1 (0.96375)

# 111 1:miRNA 1:miRNA 0.75 (0.760519)

# 112 1:miRNA 1:miRNA 1 (0.995556)

# 113 1:miRNA 1:miRNA 1 (0.984783)

# 114 1:miRNA 1:miRNA 1 (0.967882)

# 115 1:miRNA 1:miRNA 1 (0.961333)

# 116 1:miRNA 1:miRNA 1 (0.963944)

# 117 1:miRNA 1:miRNA 1 (0.938958)

# 118 1:miRNA 1:miRNA 1 (0.941461)

# 119 1:miRNA 1:miRNA 1 (0.979125)

# 120 1:miRNA 1:miRNA 1 (0.865962)

# 121 1:miRNA 1:miRNA 1 (0.946415)

# 122 1:miRNA 1:miRNA 1 (0.595429)

# 123 1:miRNA 1:miRNA 1 (0.985542)

# 124 1:miRNA 1:miRNA 1 (0.960971)

# 125 1:miRNA 1:miRNA 1 (0.914505)

# 126 1:miRNA 1:miRNA 1 (0.952529)

# 127 1:miRNA 1:miRNA 1 (0.997654)

# 128 1:miRNA 1:miRNA 1 (0.956818)

# 129 1:miRNA 1:miRNA 1 (0.947294)

# Supplementary Tables

**Table S3.** Sensitivity performance on TE-CS testing dataset of Xue et al (8)

| TE-CS | No. of  Pre-miRNAs | yasMir  (%) | Triplet-SVM (%) | PmirP  (%) | mirExplorer  (%) | Our Method(%) |
| --- | --- | --- | --- | --- | --- | --- |
| Mus musculus | 36 | 97.2 | 94.4 | 94.4 | 97.2 | 100 |
| Rattus norvegicus | 25 | 84 | 80 | 92 | 80 | 96 |
| Gallus gallus | 13 | 100 | 84.6 | 100 | 100 | 100 |
| Danio rerio | 6 | 83.3 | 66.7 | 83.3 | 83.3 | 100 |
| Caenorhabditis briggsae | 73 | 100 | 95.9 | 97.3 | 95.8 | 98.6 |
| Caenorhabditis elegans | 110 | 92.7 | 86.4 | 86.4 | 83.6 | 95.4 |
| Drosophila pseudoobscura | 71 | 94.3 | 90.1 | 91.5 | 91.5 | 97.1 |
|  |  |  |  |  |  |  |
| Drosophila melanogaster | 71 | 95.7 | 91.5 | 95.8 | 90.1 | 98.5 |
| Oryza sativa | 96 | 96.8 | 94.8 | 100 | 100 | 100 |
| Arabidopsis thaliana | 75 | 97.3 | 92 | 96 | 96 | 98.6 |
|  |  |  |  |  |  |  |
| Epstein Barr virus | 5 | 80 | 100 | 80 | 100 | 100 |
| Total | 581 | 95.3 | 90.9 | 94 | 92.4 | 98.1 |

**Table S4.** The performance on 16 species in the “*CROSS-SPECIES*” test data of miRExplorer (58)

| Cross species test set | No. of  pre-miRNAs | mirExplorer  (%) | Our Method (%) |
| --- | --- | --- | --- |
| Caenorhabditis briggsae | 94 | 92.55 | **96.80** |
| Caenorhabditis elegans | 151 | 92.05 | **96.68** |
| Bos taurus | 112 | 96.43 | **98.21** |
| Ciona intestinalis | 34 | **85.29** | 79.41 |
| Drosophila melanogaster | 144 | 93.06 | **97.22** |
| Drosophila pseudoobscura | 71 | 97.18 | 97.18 |
| Danio rerio | 336 | 95.54 | **96.72** |
| Rattus norvegicus | 277 | 95.31 | **98.55** |
| Schmidtea mediterranea | 63 | 93.65 | **96.82** |
| Xenopus tropicalis | 182 | 97.80 | 97.80 |
| Arabidopsis thaliana | 144 | 96.53 | **100** |
| Chlamydomonas reinhardtii | 42 | **100** | 95.23 |
|  |  |  |  |
| Oryza sativa | 293 | 97.61 | **99.65** |
| Epstein Barr virus | 23 | 95.65 | **100** |
| Human cytomegalovirus | 11 | 100 | 100 |
|  |  |  |  |
| Rhesus lymphocryptovirus | 16 | 100 | 100 |
| Total | 1993 | 95.53 | **97.64** |

**Table S5.** Summary of prediction result on cross species pre-miRNA in MiRBase version 17 and 18 (See: detailed data is in **Supplementary Data 1** (Excel File))

| **Organism** | **Version 17** | | | **Version 18** | | |
| --- | --- | --- | --- | --- | --- | --- |
|  | **number of species** | **number of pre-miRNAs** | **Correctly predict (%)** | **number of**  **species** | **number of**  **pre-miRNAs** | **Correctly predict (%)** |
| Animal | 85 | 13,169 | 92.74 | 93 | 13,972 | 92.89 |
| Plant | 46 | 3,362 | 98.04 | 52 | 4,014 | 97.38 |
| Virus | 22 | 237 | 94.09 | 23 | 240 | 94.17 |
| Total | 153 | 16,768 | 93.82 | 168 | 18,226 | 93.9 |

**Table S6.** Prediction result on experimentally verified pre-miRNAs from miRNAMap database version 2 (59)

| **Species** | **Number of pre-miRNA** | **Correctly predicted** | **% Correct** |
| --- | --- | --- | --- |
| Homo sapien | 475 | 475 | 100 |
| Mus musculus | 386 | 362 | 93.78 |
| Rattus norvegicus | 228 | 226 | 99.12 |
| Gallus gallus | 146 | 142 | 97.26 |
| Danio rerio | 349 | 337 | 96.56 |
| Xenopus tropicalis | 177 | 174 | 98.3 |
| Monodelphis domestica | 107 | 103 | 96.26 |
| Caenorhabditis elegans | 133 | 127 | 95.48 |
| Drosophila melanogaster | 78 | 76 | 97.43 |
| Anopheles gambiae | 38 | 37 | 97.36 |
| Canis familiaris | 6 | 5 | 83.33 |
| Fugu rubripes | 133 | 131 | 98.49 |
| **Total** | **2,256** | **2195** | **97.29** |

**Table S7.** Average value of MFE, SC, and SC-Base pair composite features of animal, plant, virus pre-miRNAs, and negative class data.

| Type | no. of sequences | Features | | | | |
| --- | --- | --- | --- | --- | --- | --- |
|  |  | MFE | SC | SCxdP | SC/(1-dP) | SCxdP/(1-dP) |
| **Pre-miRNA:** |  |  |  |  |  |  |
| Human | 1,424 | -39.84±12.14 | 0.86±0.18 | 0.31±0.08 | 1.36±0.32 | 0.50±0.16 |
| Animals | 11,746 | -35.59±10.50 | 0.85±0.17 | 0.30±0.07 | 1.32±0.29 | 0.46±0.13 |
| Plants | 3,362 | -66.32±36.37 | 0.90±0.15 | 0.32±0.07 | 1.42±0.27 | 0.51±0.14 |
| Viruses | 237 | -38.28±13.15 | 0.87±0.16 | 0.31±0.07 | 1.36±0.27 | 0.50±0.13 |
| **Negative data:** |  |  |  |  |  |  |
| ncRNA | 12,387 | -33.16±24.17 | 0.59±0.19 | 0.17±0.07 | 0.83±0.30 | 0.24±0.11 |
| Shuffle | 21,470 | -27.14±17.60 | 0.40±0.14 | 0.11±0.04 | 0.56±0.20 | 0.15±0.06 |
| Pseudo | 4,494 | -26.69±8.09 | 0.44±0.18 | 0.13±0.05 | 0.63±0.27 | 0.18±0.08 |

# Supplementary Figures


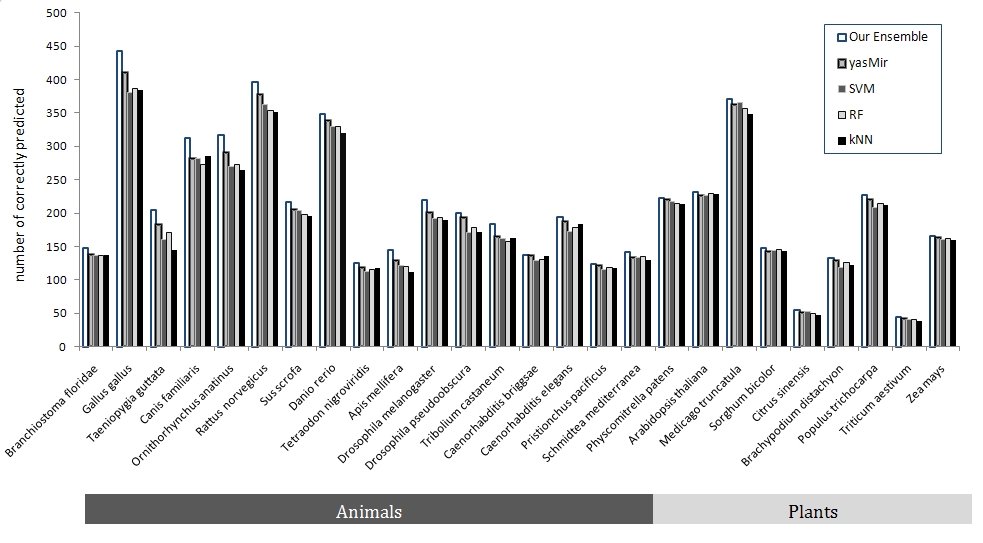


**Figure S3**: Prediction results in this study compared to single SVM, kNN, RF, and yasMir. The individual models, SVM, kNN, and RF, were built using 20 selected features from CFS+GA and trained on original imbalanced data. The yasMir is a SVM classifier using the 169 base-pairing features (12).


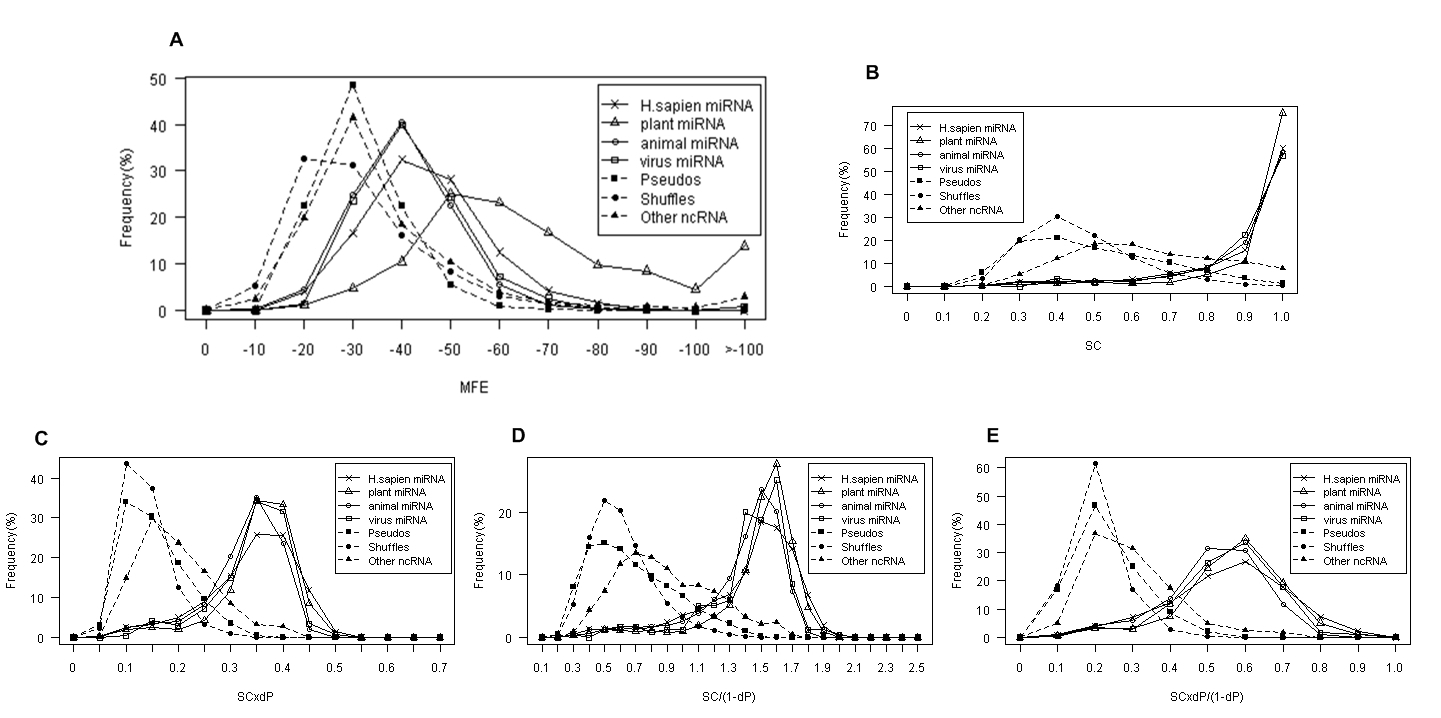


**Figure S4** The distribution of MFE, SC and SC derivative values for the testing data. (A) MFE value (B) SC value (C) SCxdP (D) SC/(1-dP) (E) SCxdP/(1-dP)

**Supplementary References**

# 62. Zuker,M. (2003) Mfold web server for nucleic acid folding and hybridization prediction. *Nucl. Acids Res.*, 31, 3406–3415.

# 63. McCaskill,J.S., (1990) The equilibrium partition function and base pair binding probabilities for RNA secondary structures. *Biopolymers*, 29, 1105-1119.

# 64. Hofacker,I.L., Fontana,W., Stadler,P.F., Bonhoeffer,S., Tacker,M. and Schuster,P. (1994) Fast folding and comparison of RNA secondary structures. *Monatshefte f. Chemie,* 125, 167-188.

# 65. Zuker,M. and Stiegler,P. (1981) Optimal computer folding of large RNA sequences using thermodynamic and auxiliary information. *Nucl. Acids Res.*, 9, 133-148.

# 66. Hofacker,I.L. (2003) Vienna RNA secondary structure server. *Nucl. Acids Res.*, 31, 3429-3431.

# 67. Zhang,B., Pan,X., Cox,S., Cobb,G., and Anderson,T. (2006) Evidence that miRNAs are different from other RNAs. *Cell. Mol. Life Sci.,* 63, 246–254.

# 68. Bonnet,E., Wuyts,J., Rouze,P., Van de Peer,Y. (2004) Evidence that microRNA precursors, unlike other non-coding RNAs, have lower folding free energies than random sequences. *Bioinformatics*, 20, 2911–2917.

69. Freyhult,E., Gardner,P., and Moulton,V. (2005) A comparison of RNA folding measures**.** *BMC bioinformatics*, **6**, 241.

70. Tempel,S. and Tahi,F. (2012) A fast ab-initio method for predicting miRNA precursors in genomes. *Nucl. Acids Res.*, **40**, e80.

71. Tyagi,S., Vaz,C., Gupta,V., Bhatia,R., Maheshwari,S., Srinivasan,A. and Bhattacharya,A. (2008)

CID-miRNA: a web server for prediction of novel miRNA precursors in human genome. *Biochem. Biophys. Res. Comm.*,**372**, 831-834.

72. Wu,Y., Wei,B., Liu,H., Li,T. and Rayner,S. (2011) MiRPara: a SVM-based software tool for prediction of most probable microRNA coding regions in genome scale sequences.*BMC Bioinformatics*,**12**, 107.

73. Grundhoff,A., Sullivan,C.S. and Ganem,D.A. (2006) Combined computational and microarray-based approach identifies novel microRNAs encoded by human gamma-herpesviruses. **RNA**, *12,* 733-750.
